# Appropriate tube temperature for fiberoptic bronchoscope-guided intubation of thermally softened double-lumen endotracheal tubes: A CONSORT-compliant article

**DOI:** 10.1097/MD.0000000000029999

**Published:** 2022-10-07

**Authors:** Yang Yu, Qianqian Jia, Lijie Zhou, Zhou Liu, Shujuan Liang, Zhen Yang, Qiong Wan

**Affiliations:** a Department of Anesthesiology, The First Hospital of Qinhuangdao City, Qinhuangdao, Hebei, China; b Research and Development Center, Contec Medical Systems Co. Ltd., Economic and Technological Development Zone, Qinhuangdao, Hebei, China; c Department of Health Services, the Third Medical Center of Chinese PLA General Hospital, Beijing, China.

**Keywords:** double-lumen endotracheal tube, fiberoptic bronchoscope, intubation resistance monitoring, thermal softening

## Abstract

To compare the effects of thermal softening of double-lumen endotracheal tubes (DLT) at different temperatures during fiberoptic bronchoscopy (FOB)-guided intubation.

**Methods:**

We randomly divided 144 patients undergoing thoracic surgery into 4 groups as follows: T1 (T = 24 ± 1°C, n = 36), T2 (T = 36 ± 1°C, n = 36), T3 (T = 40 ± 1°C, n = 36), and T4 (T = 48 ± 1°C, n = 36). All groups underwent FOB-guided double-lumen endotracheal intubation and positioning. We recorded the duration of positioning and intubation using DLT, intubation resistance (IR), the success rate of the first attempt at endotracheal intubation, and the incidence of postoperative vocal cord injury and hoarseness.

**Results:**

The time to intubation was longer in the T1 group than that in the T2, T3, and T4 groups (*P* < .05). The time for positioning was longer in the T4 group than that in the T1, T2, and T3 groups (*P* < .05). IR was lower in the T3 and T4 groups than those in T1 and T2 groups (*P* < .05). The success rate of the first attempt at endotracheal intubation was higher in the T2, T3, and T4 groups than that in the T1 group (*P* < .05). Postoperative glottic injury and hoarseness were higher in the T1 and T2 groups than those in the T3 and T4 groups (*P* < .05).

**Conclusion:**

A thermally softened DLT shortened the time to intubation, reduced the IR, improved the success rate of the first attempt at endotracheal intubation, and lowered the incidence of postoperative glottic injury and hoarseness. The optimal tube temperature for FOB-guided intubation of thermally softened DLT was 40 ± 1°C.

## 1. Introduction

One-lung ventilation (OLV) is principally required for thoracic surgeries, and double-lumen endotracheal tubes (DLTs) are the commonly used devices. Compared with the traditional intubation of DLT via laryngoscopes, fiberoptic bronchoscope (FOB)-guided intubation of DLT can reduce the time for positioning and the incidence of malpositioning,^[1,2]^ which is however difficult to operate. Currently, polyvinyl chloride (PVC) is the most commonly used DLT material. Compared with a single tracheal tube, DLTs with a large diameter, longer tube length, harder texture, and a sharp tip lead to greater resistance when the tube passes through the glottis. Problems, such as poor tube delivery, may cause laryngotracheal injury and hoarseness.^[3]^ Therefore, FOB-guided intubation of the DLT is challenging.

The intubation of thermally softened PVC DLTs is easy, similar to that of flexible silicone tracheal tubes.^[4]^ Thermally softened DLTs can reduce the incidence of sore throat and vocal cord injury postoperatively.^[5–8]^ PVC DLTs demonstrate good flexibility at 60°C following thermal softening treatment, without causing burns. Moreover, for endotracheal intubation without burns, the surface temperature should be kept below 50°C.^[9]^ However, researchers have not yet determined the appropriate temperature for FOB-guided intubation with thermally softened DLTs. Therefore, we aimed to compare the effects of thermal softening of DLTs at different temperatures during FOB-guided intubation.

## 2. Patients and Methods

### 2.1. Study population

This study was approved by the Medical Ethics Committee of the First Hospital of Qinhuangdao City Affiliated to the Hebei Medical University (Approval Number: 2019D004), and written informed consent was obtained from all patients. We selected 144 patients aged 20 to 70 years, with American Society Anesthesiologists physical status I-II, Cormack-Lehane (C/L) grade I-II, requiring intubation, and with left-sided DLT. The exclusion criteria were as follows: tracheal distortion and lesions examined by preoperative bronchoscopy and CT, difficult mask ventilation, unpredictable difficult airway, hoarseness, sore throat, and other symptoms before surgery.

### 2.2. Intubation

All patients were evaluated and screened by a senior anesthesiologist before surgery and randomly divided into 4 groups as follows: T1 (T = 24 ± 1°C, n = 36), T2 (T = 36 ± 1°C, n = 36), T3 (T = 40 ± 1°C, n = 36), and T4 (T = 48 ± 1°C, n = 36). They were numbered according to a random number table and the randomization results were sealed in an envelope, which was opened before the experiment. In addition, the patients were grouped according to random numbers.

**Figure 1.**
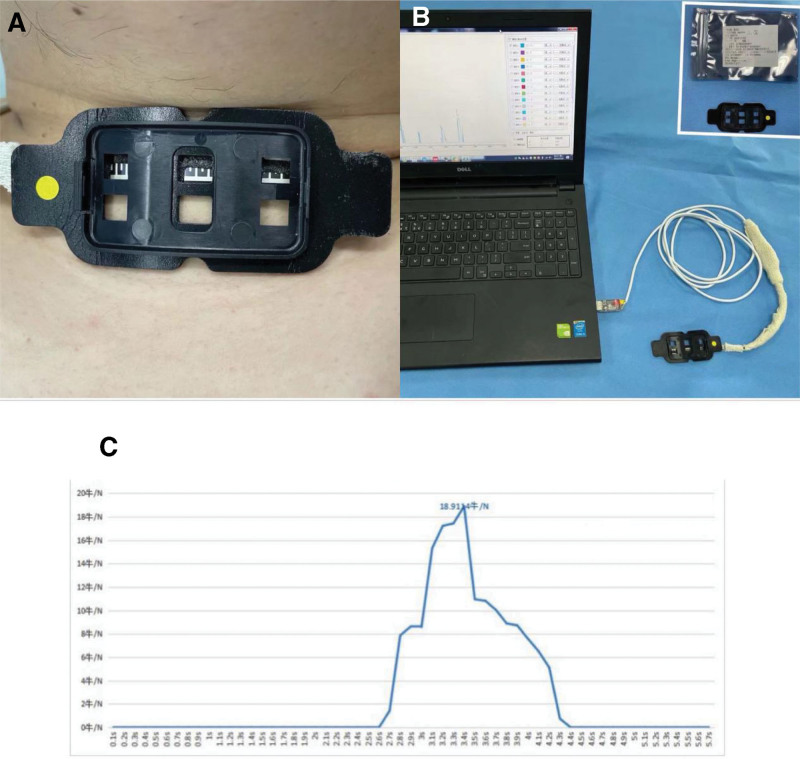
Fiberoptic bronchoscope-guided thermally softened double-lumen endotracheal tube.

**Figure 2.**
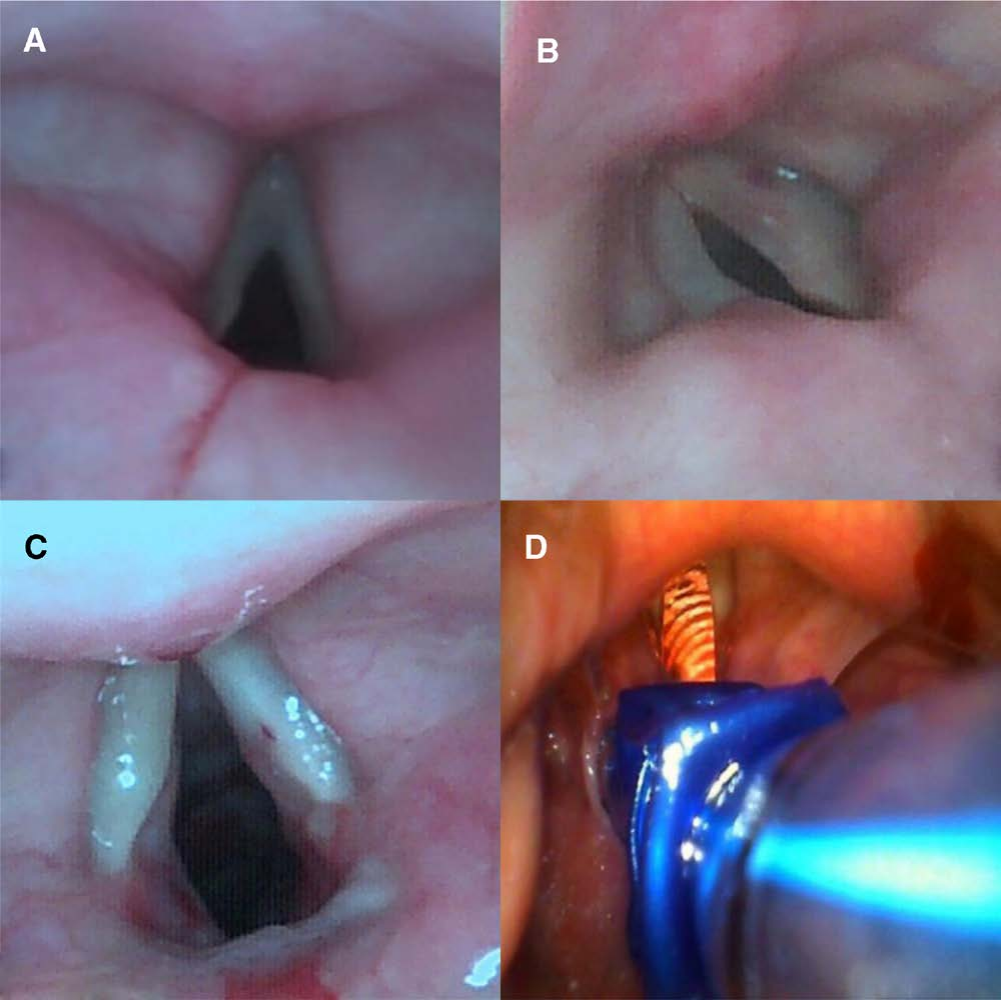
Intubation resistance detector.

**Figure 3.**
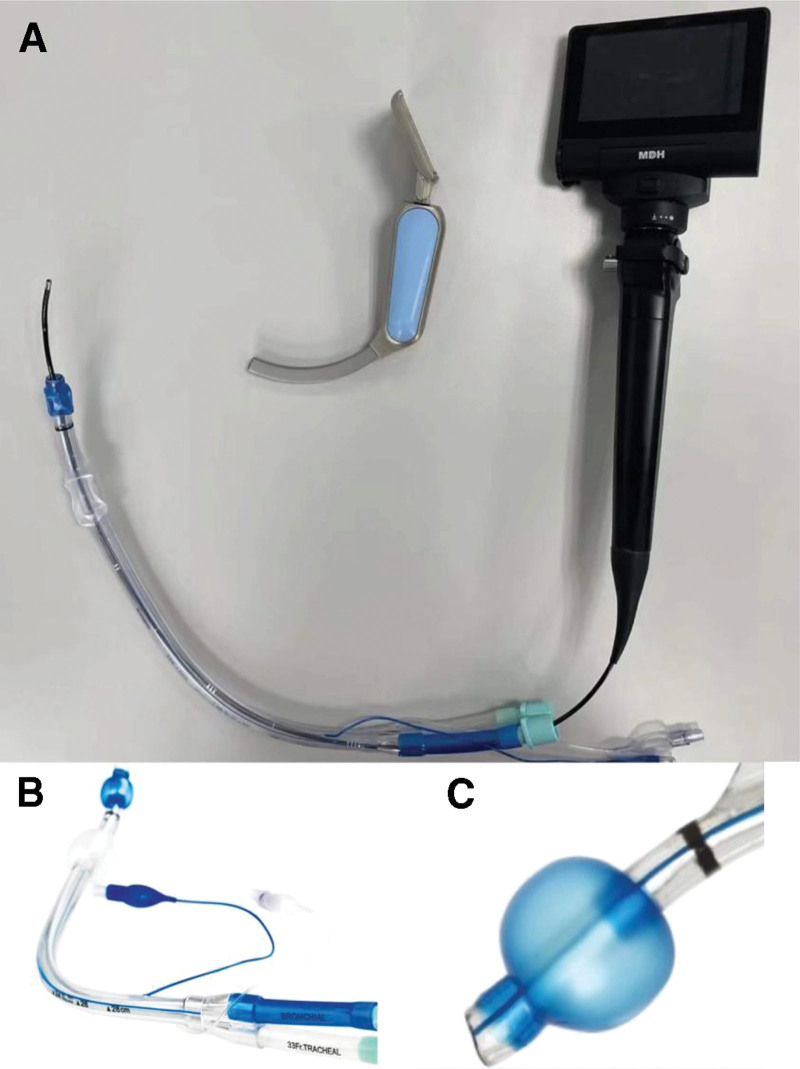
Determination of the heating temperature of the double-lumen endotracheal tube.

We placed a disposable left-sided DLT (Tuoren Medical Equipment Co., Ltd., Henan, China, Fig. 1B) in a 60°C incubator (MIR-162) for 30 minutes, and used an infrared noncontact thermometer (TH838S, Kunshan Thermal Photoelectric Co. Ltd. Jiangsu, China) to measure its temperature. We performed 70 measurements to identify changing patterns in the heating temperature and time (Fig. 2). The tube wall temperature was 50 ± 2°C immediately following tube removal, which decreased to 46.5 ± 1°C, 37.2 ± 1°C, and 24 ± 1°C at 30 seconds, 60 seconds, and 180s post tube removal, respectively. The time to intubation in the T2 group was 62 ± 1 second following tube removal, and the temperature was controlled at 36 ± 1°C. The time to intubation in the T3 and T4 group was 44 ± 1 and 33 ± 1 seconds following tube removal, and the tube temperature was controlled at 40 ± 1°C and 48 ± 1°C, respectively (Fig. 3). The similar senior anesthesiologist performed endotracheal intubation to avoid discrepancy owing to errors or differences in the operating techniques.

All patients received intramuscular midazolam (0.05 mg/kg) at 30 minutes before entering the operating room. Following their entry, we recorded the noninvasive blood pressure, electrocardiogram, heart rate, pulse oxygen saturation, and disposable bispectral index sensor (BIS). We placed the intubation resistance (IR) detector below the thyroid cartilage (projection of the glottis on the body surface) (Fig. 2A).

Lidocaine (40 mg), propofol (2 mg/kg), rocuronium (0.6 mg/kg), and sufentanil (1 μg/kg) were intravenously injected. For a BIS value reduced to 50, we maintained anesthesia with target-controlled infusion of propofol (6 μg/mL). Furthermore, the aforementioned anesthesiologist evaluated the C/L grade using a direct laryngoscope. The patients were placed in a supine position, following which an anesthesia assistant provided continuous positive airway pressure. In the T2, T3, and T4 groups, the thermally softened DLT was removed from the incubator and measured using an infrared thermometer. Upon reaching the target temperature of each group, we immediately performed intubation. The DLT was directly used in the T1 group. We inserted the lubricated FOB (MDH, A50, 2.8 mm, Zhuhai Maidehao Medical Technology Co, Ltd, Guangzhou, China) through the opening of the left-sided bronchial tube and guided DLT into the glottis along the midline (Fig. 1A). We recorded the time to intubation. FOB-guided intubation of the DLT was performed along the lingual surface through the glottis into the trachea, and the time for positioning was recorded. Following an arriving at the tracheal carina, the lens entered the left mainstem bronchus. We removed the FOB upon the entry of the distal end of the DLT into the left mainstem bronchus. The FOB was inserted into the right bronchial tube and removed after determining the position of the DLT, thereby leading to the correct positioning of the DLT. We turned on the control of the ventilator machine and observed the end-tidal CO_2_ and airway pressure, followed by auscultation of both lungs and completed positioning. Endotracheal intubation was performed by the similar anesthesiologist. During the operation, propofol, remifentanil, and rocuronium were continuously administered. Following extubation, we observed the glottis with FOB and recorded the results by photography. Moreover, we recorded the occurrence of sore throat and hoarseness at 24 hours postsurgery. To overcome the subjectivity of evaluation, we selected different anesthesiologists for the double-blind follow-up, and both the anesthesiologists and patients were blinded to the patient grouping. Direct laryngoscopy grading was conducted by the similar physician who performed endotracheal intubation.

We defined a failure in intubation as the inability to insert the DLT into the left mainstem bronchus following 2 attempts.

**Figure 4.**
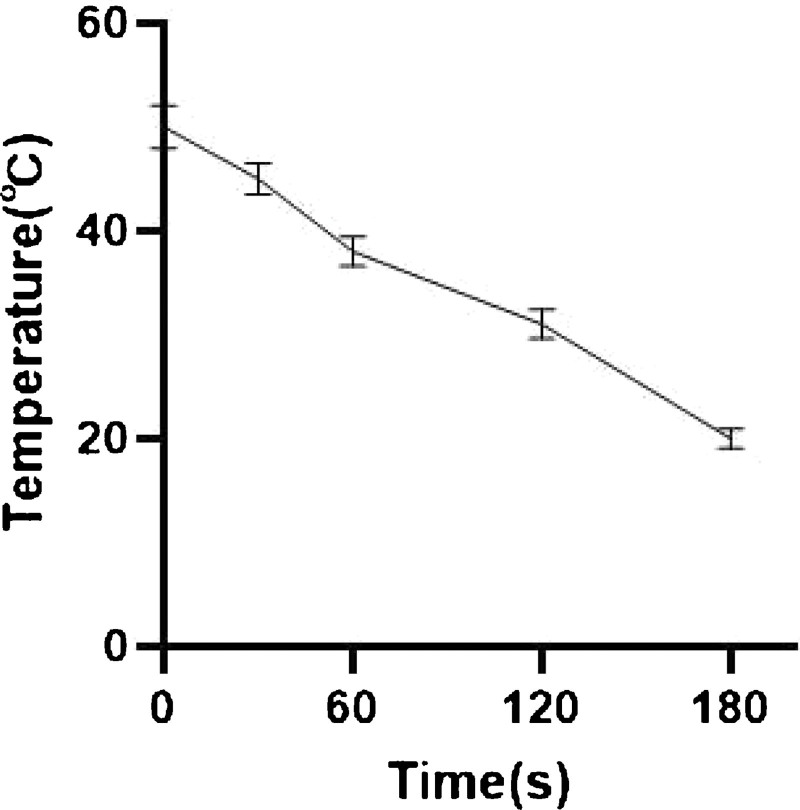
Grading of glottic injury before intubation and immediately after extubation.

The primary outcomes were the intubation time (IT) calculated from the entry of the distal end of the FOB into the oral cavity to the cuff of the DLT through the glottis and glottic injury observed by FOB before and immediately after extubation. These outcomes were photographed for comparison. We classified glottic injury into mild (mucosal edema and hyperemia) (Fig. 4A), moderate (mucosal erythema and hemorrhage) (Fig. 4B), and severe types (mucosal granuloma) (Fig. 4C).

The secondary outcomes included the time for positioning calculated from the time of the entry of the cuff of the DLT into the glottis to the correct positioning, IR, the success rate of the first attempt at endotracheal intubation, and the incidence of postoperative hoarseness. An IR detector (Fig. 2A) was placed below the thyroid cartilage. The output resistance decreased with increased pressure on the sensor surface. According to the specific pressure-resistance relationship, IR could be calculated from the resistance (Fig. 2C).

### 2.3. Statistical analysis

We randomized the difference in time to the intubation of the DLT at the 4 temperatures. According to the pre-experiment, the mean time to intubation in the T1, T2, T3, and T4 groups was predicted to be 45 ± 7, 36 ± 6, 33 ± 6, and 34 ± 5 seconds, respectively. A bilateral test was required; moreover, the significance and test effectiveness was set at 0.05% and 80%, respectively. The sample size required to detect differences in the time to intubation was 120 patients. Considering factors, such as the failure of intubation and refusal to follow-up, we selected 144 patients (n = 36 per group).

We used the SPSS 21 statistical software for the data analysis. Continuous variables are expressed as mean ± standard deviation. We performed an independent-sample t-test and paired-sample t-test for the inter- and intragroup differences, respectively. The chi-square test was performed to determine the proportional differences. Moreover, we conducted the Fisher exact test to analyze differences in the success rate of the first attempt at endotracheal intubation and the incidence of hoarseness. The statistical significance was set at *P* < .05.

## 3. Results

**Figure 5.**
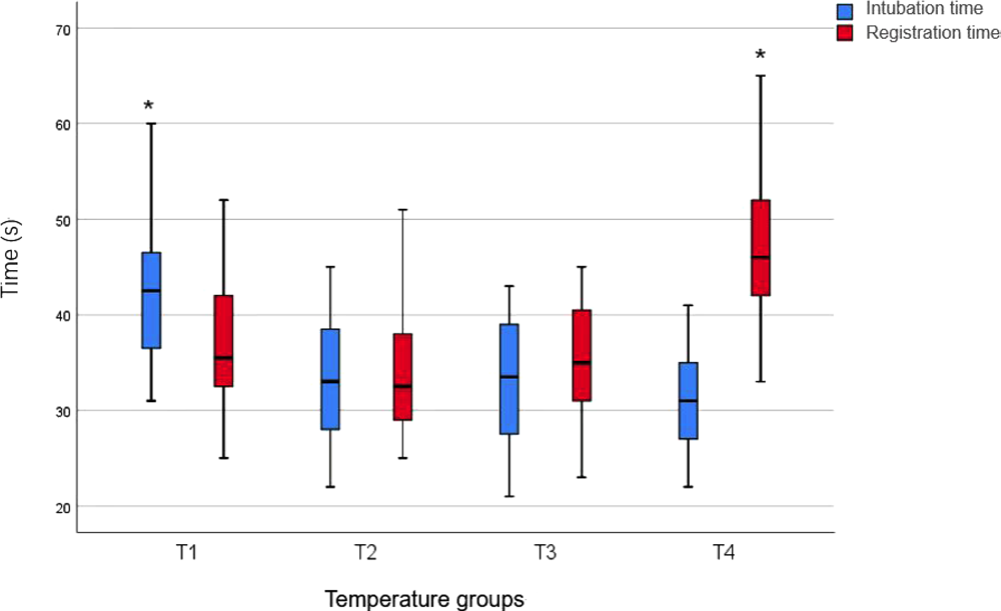
The time to intubation and the time for positioning.

From December 2019 to March 2021, all patients underwent thoracic surgeries and were randomly divided into 4 groups, without significant differences in general conditions (Table 1). The time to intubation was significantly longer in the T1 group than that in the T2, T3, and T4 groups (*P* < .05) (Table 2; Fig. 5). Postoperative glottic injury in the T1 and T2 groups was higher than that in the T3 and T4 groups (*P* < .05) (Table 2). The time for positioning was longer in the T4 group than that in the T1, T2, and T3 groups (*P* < .05) (Table 2; Fig. 5). The highest IR was observed in the T1 group (38.57 N), whereas IR was lower in the T3 and T4 groups than that in T1 and T2 groups (*P* < .05) (Table 2). The success rate of the first attempt at endotracheal intubation was higher in the T2, T3, and T4 groups than those in the T1 group (*P* < .05). Moreover, there was no statistical difference among the T2, T3, and T4 groups (Table 2). The incidence of postoperative hoarseness was higher in the T1 and T2 groups than that in the T3 and T4 groups (*P* < .05) (Table 2).

**Table 1:** General conditions of patients in the 4 groups.

	T_1_ (n = 36)	T_2_ (n = 36)	T_3_(n = 36)	T_4_(n = 36)	*P*
Age(mean ± SD, y)	55.8 ± 8.8	58.5 ± 8.8	55.3 ± 9.5	57.6 ± 9.3	.39
Sex (n, M/F)	14/22	28/8	18/18	25/11	
Height (mean ± SD, cm)	164.1 ± 7.1	165.1 ± 6.2	166.2 ± 6.6	165.6 ± 7.2	.61
Weight (mean ± SD, kg)	64.9 ± 8.7	64.6 ± 8.5	66.3 ± 9.4	65.2 ± 8.5	.85
BMI (mean ± SD, kg/m^2^)	24.0 ± 6.3	23.8 ± 3.3	23.9 ± 2.8	23.7 ± 2.4	.98
ASA(I/II)	11/25	17/19	16/20	13/23	.47
Cormack-Lehane score					.13
I	10	8	6	12	
II*	12	15	22	18	
II†	14	13	8	6	

Statistical analysis indicated no significant differences in the parameters among the groups (*P* > .05).

ASA = American Society of Anesthesiologists, BMI = body mass index.

*Partial view of glottis visible.

†Only the arytenoids visible.

**Table 2:** Comparison of the time to intubation, the time for positioning, intubation resistance, the success rate of the first attempt at endotracheal intubation, and the incidence of postoperative hoarseness and glottic injury.

	T_1_ (n = 36)	T_2_ (n = 36)	T_3_ (n = 36)	T_4_ (n = 36)	*P*
Time to intubation (mean ± SD, s)	42.1 ± 7.5*	33.4 ± 6.6	32.6 ± 6.4	30.7 ± 5.3	<.01
Time for positioning (mean ± SD, s)	37.2 ± 6.8	34.4 ± 6.6	35.4 ± 5.6	46.6 ± 7.2*	<.01
Intubation resistance (mean ± SD, N)	32.7 ± 3.7*	27.6 ± 3.4*	17.2 ± 2.3	17.8 ± 2.2	<.01
Successful of first intubation (n, %)	26 (72.2%)*	31 (86.1%)	35 (97.2%)	33 (91.7%)	.02
Hoarseness (n, %)	11 (30.6%)*	9 (25.0%)*	5 (13.9%)	3 (8.3%)	.04
Glottic injury (n, %)	18 (50%)*	15 (41.7%)*	7 (19.4%)	5 (13.9%)	<.01
Mild	8	9	6	4	
Moderate	8	5	1	1	
Severe	2	1	0	0	

* *P* < .05, compared with the other 3 groups.

## 4. Discussion

Thermal softening of the DLTs above 40°C led to shorter time to intubation and less postoperative glottic injury. The optimal temperature for FOB-guided intubation of DLTs was 40 ± 1°C.

The advantage of FOB-guided intubation using DLTs is that it enables simultaneous intubation and positioning, thus resulting in the correct placement of the DLT in a shorter period.^[1]^ However, the procedure can be challenging, particularly to beginners. This is principally attributed to the larger diameter, hard texture, and poor flexibility of the DLT. Despite the successful insertion of FOB into the airway, the front end of the DLT was deviated from the FOB and moved toward the epiglottis, arytenoid cartilage, piriform sinus, and esophagus upon reaching the larynx; thus, it was blocked in the larynx, thereby leading to the failure of DLT advancement into the airway.^[4,10]^ This would not only prolong the time to intubation but also damage the vocal cords and surrounding tissues.^[11]^ Following thermal softening, the PVC tube could reduce the difficulty of endotracheal intubation, shorten the time to intubation, and reduce the laryngeal injury caused by intubation.^[6,12,13]^

**Figure 6.**
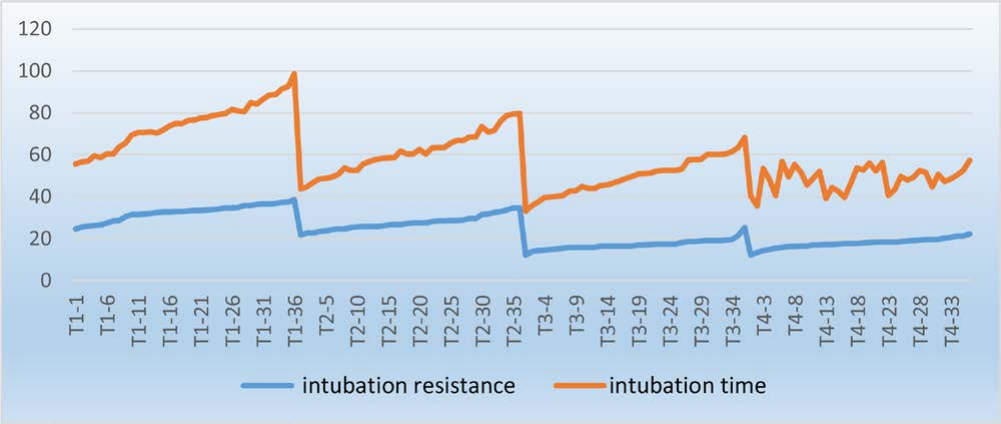
Changing trend of intubation resistance and the time to intubation.

The mean time to intubation was 42.14 seconds in the T1 group, which was considerably longer than that in the T2, T3, and T4 group (33.44 seconds, 32.61 seconds, and 30.69 s, respectively, Table 1). The time to intubation gradually decreased with increased DLT temperature (Fig. 6). According to Dr Ji Young Yoo, FOB-guided intubation using silicone double-lumen endotracheal tube was rapid, similar to that using a single-lumen tube. The flexible, wire-reinforced bronchial tip enabled a rapid change in the silicone DLT, thereby following the movement of FOB.^[14]^ FOB-guided flexible tubes entered the airway more easily than PVC tubes.^[15,16]^ A simulation study demonstrated that soft silicone DLTs with flexible, wire-reinforced bronchial ends (Fuji-Phycon tubes) significantly reduced the time to intubation, compared with PVC DLTs, such as Mallinckrodt or Rusch DLT.^[17]^ In the present study, the DLT used had flexibility similar to silicone DLT following thermal softening treatment. Its hardness decreased proportionally with an increase in temperature,^[10]^ thus resulting in soft and flexible walls, which fitted better with the FOB. Moreover, it revealed sufficient flexibility and bent with the FOB. The DLT in the thermal softening group (T2, T3, and T4 groups) passed through the glottis more easily than that in the T1 group. Higher temperature was associated with better flexibility and shorter time to intubation. The mean time for positioning in the T4 group was 46.58 seconds, which was longer than that in the T1, T2, and T3 groups (37.2 seconds, 34.42 seconds, and 35.44 seconds, respectively). Three patients in the T4 group revealed that the tip of the DLT was stuck in the carina following the DLT insertion into the left bronchus over the FOB, whereas the tip of the right bronchus could not be inserted into the left bronchus. Of these patients, 2 experienced the withdrawal of the tube to the subglottic area and an adjustment of the direction of the tube to enter the left bronchus, successfully guided by FOB; however, 1 patient failed. Enda Shanahan et al reported that following tube softening at 21°C, 40°C, 45°C, and 50°C, the end of the DLT was rotated 180° counter-clockwise and its tip was rotated 119°, 119°, 92°, and 53° counter-clockwise, respectively, during intubation.^[12]^ Accordingly, for higher thermal temperature of the DLT, the wall of the DLT would be considerably soft to rotate it to the target bronchi. The tip of the DLT may be stuck in the carina upon entering the left major airway. Therefore, the DLT in the T4 group was not the optimal option, and may unnecessarily expose patients to risks, such as thermal injury and difficult intubation.

**Figure 7.**
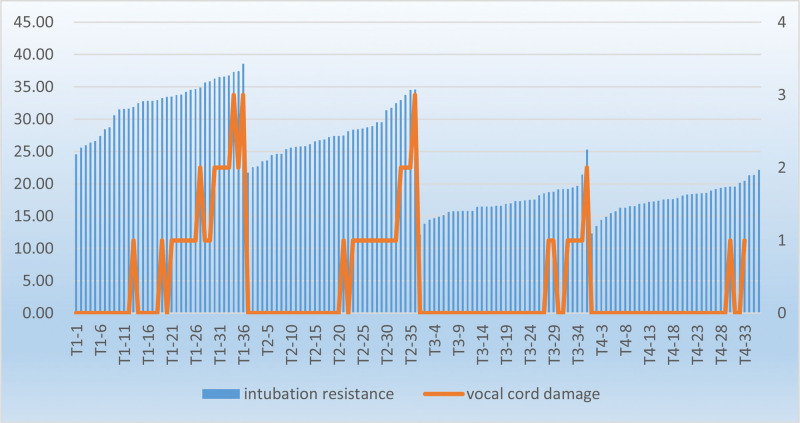
Changing trend of intubation resistance and vocal cord injury degree.

Postoperative sore throat and hoarseness are common complications following intubation of the DLT. There are several methods to reduce postoperative pharyngeal injury caused by endotracheal intubation.^[18–20]^ They are associated with reduced incidence of postoperative sore throat, thus suggesting the irritation of airway tissues during intubation may be a major cause of complications induced by the intubation of the DLT.^[21]^ Dr J-H Seo used warm saline to heat DLT for endotracheal intubation, and reported that endotracheal intubation of thermally softened DLT could reduce the incidence of postoperative sore throat and vocal cord injury.^[6]^ This was consistent with our findings that glottic injury caused by endotracheal intubation was less in the T3 and T4 groups than that in the T1 and T2 groups. In addition, heating above 40°C could reduce glottic injury caused by intubation. According to Dr Seo, thermally softened DLT traveled more smoothly through the glottis with lower resistance and fewer bleeding spots, thus suggesting less vocal cord injury.^[6]^ However, some scholars argue that Dr Seo did not provide a standard for the resistance generated owing to the entry of the DLT into the glottis, besides the evaluation of resistance being subjective. In addition, they raised a question if all glottal resistance could be attributed to the impact of DLT on the glottis, for example, if DLT intubation using a direct laryngoscope could produce resistance.^[22]^ In this study, we used a resistance detector (R&D Department of Kangtai Medical Co., Ltd.) to measure the resistance caused by the DLT while passing through the glottis. Any part of the airway may get damaged during intubation; nonetheless, the vocal cords are the most vulnerable because the glottis is the narrowest part in close contact with the trachea.^[23]^ We placed the resistive pressure sensor below the thyroid cartilage, and the output resistance decreased with increased pressure on the sensor surface (See Figure 1, Supplemental Digital Content, which illustrates tube pressure-resistance conversion, http://links.lww.com/MD/H10) According to the specific pressure-resistance relationship, we calculated the pressure of the tube on the glottis, which directly reflected the pressure of the DLT on the glottis (See Figure 2, Supplemental Digital Content, which illustrates collection of electrical pressure values, http://links.lww.com/MD/H11; See Figure 3, Supplemental Digital Content, which illustrates relationship between electrical pressure values and pressure values, http://links.lww.com/MD/H12). The mean IR was 32.65 N and 27.59 N in the T1 and T2 groups, respectively, which were higher than those in the T3 and T4 groups (17.15 N and 17.79 N, respectively). The IR reduced with the increase in DLT temperature. Similarly, the incidence of postoperative glottic injury and hoarseness was higher in the T1 and T2 groups than that in the T3 and T4 groups, which was correlated with the IR. The degree of glottic injury of patients in each group increased with IR (Fig. 7). We observed the highest value of IR (38.57 N, 37.86 N) in 2 patients with severe glottic injury in the T1 group. The IR was presumably a direct indicator for the difficulty of endotracheal intubation, besides being responsible for postoperative intubation-related complications. This result verified Dr Seo statement that the thermally softened DLT had soft texture, which reduced its impact on the glottis and the IR, thereby lowering the incidence of glottic injury and hoarseness. In our study, FOB-guided intubation of the DLTs did not generate resistance produced by direct laryngoscopy in the trial by Dr Seo. An anesthesiologist with 15 years of clinical experience in anesthesia performed all intubations.

In addition, of the 19 patients with a failed first intubation attempt, 10 patients reported that the DLT was unable to enter the airway owing to the obstruction of the tube in the larynx, including 9 (25%), 3 (8.3%), 1 (2.8%), and no patients in the T1, T2, T3, and T4 groups, respectively. Using video laryngoscopy, the entry of FOB in the airway blocked the leading end of the DLT in the cricoid cartilage (Fig. 4D), thereby causing IR and the failure to pass through the glottis. This finding confirmed the previous viewpoint that the tube tended to move toward the back of the glottis, which was the primary reason for the difficulty of FOB-guided peroral intubation. Following its entry into the arytenoid cartilage or the esophagus entrance, the right arytenoid cartilage was more likely to block the passage of the tube than the left arytenoid cartilage. Dr Asai T attributed it to the bevel of the tube tip.^[4]^ However, the tip of DLT was rounded and blunt (Fig. 1C), without a bevel; therefore, endotracheal tube obstruction occurred in the arytenoid cartilage on both sides (73% on the left and 27% on the right). Moreover, the probability of tube obstruction decreased with an increase in endotracheal tube temperature, thus indicating the use of thermally softened DLT was principally responsible for the passage of the tube through the glottis.

This study had several limitations. First, the IR detector was limited to the glottis, and we did not obtain results for the resistance and injury in other parts. Second, the evaluation of postoperative sore throat and vocal cord injury was subjective. Other operations, such as extubation, oral sputum aspiration, and direct laryngoscopy grading, can also cause postoperative sore throat and vocal cord injury.^[24]^ Third, video laryngoscope assistance was not performed by different anesthesia assistants, which may have affect the exposure of the glottis. We selected 3 anesthesia assistants to randomly select the cases.

## 5. Conclusions

In conclusion, the optimal temperature for FOB-guided intubation of thermally softened DLTs was 40 ± 1°C, which can facilitate faster intubation and reduce the incidence of postoperative glottic injury with good operability. Therefore, FOB-guided intubation is feasible for clinical application.

## Acknowledgments

We acknowledge the Pubmed database for the high-quality data used in this study and Editage [http://www.editage.com] for the scientific editing of this manuscript as well as for editing and reviewing it for English language.

## Author contributions

Y.Y. and L.J.Z. collected the initial data and drafted the article. Y.Y., Q.Q.J., L.J.Z., and Z.L. completed anesthesia management. Q.Q.J., Z.L., and S.J.L. helped with article editing and modified the figures and tables. Z.Y., Z.L., and S.J.L. contributed to writing. Y.Y., Q.W., and S.J.L. reviewed and edited the article. All authors have read and approved the final article.

## Supplementary Material


