# Supplementary material for: Appropriate tube temperature for fiberoptic bronchoscope-guided intubation of thermally softened double-lumen endotracheal tubes: A CONSORT-compliant article
Source: Medicine (Baltimore). 2022 Oct 7;101(40):e29999. doi: 10.1097/MD.0000000000029999 (PMC9542834; doi:10.1097/MD.0000000000029999)
Supplement: Supplementary file 1 [file medi-101-e29999-s001.docx]

**Supplemental Figure 1:** Tube pressure-resistance conversion


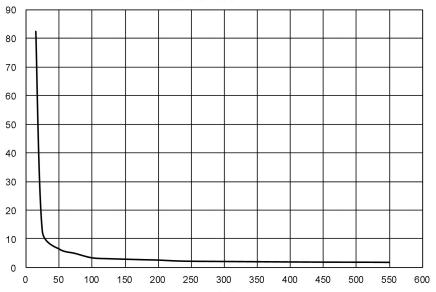


[resistance](C:/Program%20Files/WindowsApps/NeteaseYoudao.18692F27B7C6F_0.0.7.0_x64__7x355j7kq8bfj/VFS/Local%20AppData/youdao/dict/Application/0.0.7.0/resultui/html/index.html#/javascript:;)(kΩ) (kΩ)

pressure(g)

The tube pressure signal is converted to a resistance signal (Supplemental Figure 1).
