# Supplementary material for: Appropriate tube temperature for fiberoptic bronchoscope-guided intubation of thermally softened double-lumen endotracheal tubes: A CONSORT-compliant article
Source: Medicine (Baltimore). 2022 Oct 7;101(40):e29999. doi: 10.1097/MD.0000000000029999 (PMC9542834; doi:10.1097/MD.0000000000029999)
Supplement: Supplementary file 2 [file medi-101-e29999-s002.docx]

**Supplemental Figure 2:** Collection of electrical pressure values


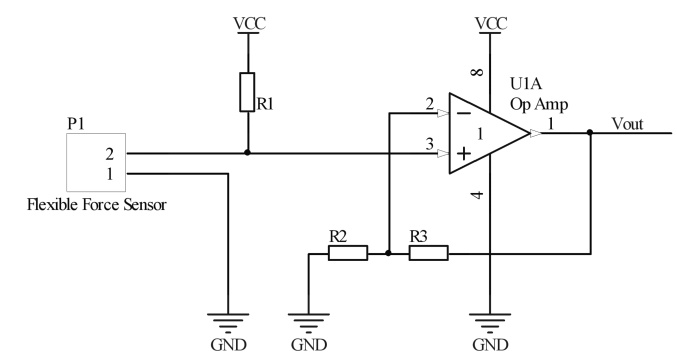


[pressure](C:/Program%20Files/WindowsApps/NeteaseYoudao.18692F27B7C6F_0.0.7.0_x64__7x355j7kq8bfj/VFS/Local%20AppData/youdao/dict/Application/0.0.7.0/resultui/html/index.html#/javascript:;) [sensor](C:/Program%20Files/WindowsApps/NeteaseYoudao.18692F27B7C6F_0.0.7.0_x64__7x355j7kq8bfj/VFS/Local%20AppData/youdao/dict/Application/0.0.7.0/resultui/html/index.html#/javascript:;)

The resistance is converted into an electrical force value (Vout) through an electrical pressure conversion device (Supplemental Figure 2), followed by calculating the pressure value on the sensor surface.
