# Supplementary material for: Appropriate tube temperature for fiberoptic bronchoscope-guided intubation of thermally softened double-lumen endotracheal tubes: A CONSORT-compliant article
Source: Medicine (Baltimore). 2022 Oct 7;101(40):e29999. doi: 10.1097/MD.0000000000029999 (PMC9542834; doi:10.1097/MD.0000000000029999)
Supplement: Supplementary file 3 [file medi-101-e29999-s003.docx]

**Supplemental Figure 3:** Relationship between electrical pressure values (V) and pressure values (g)


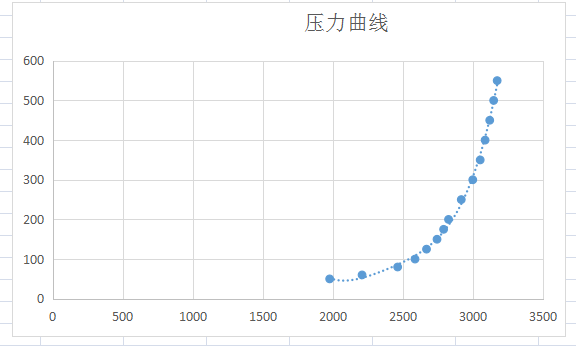


Electrical pressure (V)

pressure(g)

According to the trend diagram of the voltage value (V) and the pressure value (g) (Supplemental Figure 3), we calculated the final calculation formula of the tube pressure (N) was the curve fitting method as follows:

Pressure (g) = 8E-10×Electrical pressure (V)^4^ - 7E-06×Electrical pressure (V)^3^ + 0.0257×Electrical pressure (V)^2^ - 40.883×Electrical pressure (V) + 24426

Tube pressure (N) = pressure (g) ×0.0098

E: scientific notation, abbreviation for exponent
